# Trachoma Control: A Glass Half Full?

**DOI:** 10.4269/ajtmh.22-0760

**Published:** 2023-01-09

**Authors:** Awraris H. Bilchut, Hadley R. Burroughs, Catherine E. Oldenburg, Thomas M. Lietman

**Affiliations:** ^1^ School of Public Health, Asrat Woldeyes Health Science Campus, Debre Berhan University, Debre Berhan, Ethiopia;; ^2^ Francis I Proctor Foundation, University of California, San Francisco, San Francisco, California;; ^3^ Department of Ophthalmology, University of California, San Francisco, San Francisco, California;; ^4^ Department of Epidemiology and Biostatistics, University of California, San Francisco, San Francisco, California;; ^5^ Institute for Global Health Sciences, University of California, San Francisco, San Francisco, California

The WHO and its partners had hoped to eliminate trachoma as a public health problem by 2020. They weren’t even close. Trachoma programs have had considerable success around the world, but although 15 previously endemic countries have now declared control, about twice that many have not.^1^ Ethiopia is home to the majority of districts that have failed to meet this goal. Two publications in this issue of the *American Journal of Tropical Medicine & Hygiene* address the challenges Ethiopia faces to achieve trachoma control. The first describes Amhara, the most severely affected region in Ethiopia.^2^ Amhara is unique in that the trachoma program monitors not only clinical activity as required by WHO guidelines, but also conjunctival chlamydial infection, the cause of trachoma. The second publication reports the association of clinical trachoma with water, sanitation, and hygiene (WASH) indicators in an area of southern Ethiopia. The authors conclude that enhanced WASH may be necessary for persistent areas.^3^

The WHO decided not to set a goal of worldwide eradication of the strains of *Chlamydia* that cause trachoma, or even the lesser goal of elimination of infection regionally. Instead, it focused on the seemingly more attainable task of controlling trachoma to a low enough level that blindness would not be a public health problem. The WHO definition of control includes a prevalence of follicular trachoma below 5% in children.^4^ However, the follicles caused by trachoma take far longer to disappear than does infection itself. By the time a region reaches this threshold, evidence of infection can be difficult, if not impossible, to find. Thus ironically, achieving *elimination* may be no more difficult than *control* in this setting.

The first report highlights considerable progress against trachoma in western Amhara.^2^ Although sampling was sparse, 40% (62 of 156) of districts surveyed had no evidence of current infection. Interestingly, the majority of these districts with no evidence of infection still had not achieved control per the WHO guidelines. This confirms that *elimination of apparent infection may be an easier task than control of clinical disease*. Regardless, the vast majority of people being treated with azithromycin to control trachoma in Western Amhara are not actually infected with *Chlamydia*, the causative agent of trachoma. Presumably those areas would soon meet control targets even if mass distribution of azithromycin (MDA) were discontinued. One could argue that resources currently allocated to these districts might be better used elsewhere.

This same report tells a different story in eastern Amhara, where the majority of districts still have clinical activity well above the WHO threshold for control.^2^ Here, infection remains relatively high, with a substantial proportion of districts with > 15% prevalence of chlamydial infection. After more than a decade of MDA, progress toward elimination appears to have stalled, settling into a new equilibrium. In a number of these problem districts, the Ethiopian Ministry of Health is now considering enhanced interventions.

A popular intervention being considered is more intensive WASH. Researchers have long noted the association between WASH indicators and clinical trachoma in cross-sectional studies.^4^ In the second paper, investigators demonstrated that this relationship still holds in Ethiopia. Latrine and soap use are associated with approximately half the odds of having clinical trachoma, compared with lack of use.^3^ These authors, and many others, feel that increasing WASH efforts is the final piece necessary to achieve success. Given the strong association between WASH indicators and trachoma control, why are programs not more aggressive with WASH? The easy answer is that association is not causation. Few would disagree with the relatively strong correlation between WASH and trachoma. Yet no one has been able to prove that any particular WASH intervention has any effect on clinical trachoma or on ocular chlamydial infection.^4^ A recent community-randomized trial demonstrated that a comprehensive package that included creating water access, distributing soap, and encouraging latrine use significantly changed behavior and WASH indices. Unfortunately, the trial found no reduction in ocular chlamydial infection in those receiving the WASH intervention.^5^ Even without causal evidence, some argue that promoting WASH couldn’t hurt. No one would argue against water, latrines, and hygiene as basic instruments of public health, if not basic rights. But until we have a scientific justification for WASH, programs focused on controlling trachoma may not want to devote their limited resources to unproven, and in some cases expensive, interventions.

Like it or not, the only proven enhancement to annual antibiotics is even more antibiotics. That is, twice yearly, rather than annual distribution of azithromycin to entire communities, rather than only children, or quarterly distributions targeted to a core group of children.^6^ Models have predicted, and clinical trials have confirmed, that more-frequent-than-annual MDA should eliminate infection in even the most severely affected communities.^7^ Ethiopia has been a leader in researching more frequent MDA in trachoma-endemic communities. The Ministry of Health is now considering twice-yearly distributions to the most affected districts and is planning to monitor not just clinical activity but also tests for infection based on polymerase chain reaction and serology. One severely affected district in Amhara is currently assessing quarterly distributions.

The course of the world’s trachoma efforts will likely be dictated by what happens in Ethiopia. With current interventions alone, the worst areas may have to wait for a secular trend to eliminate infection. That could take years. Discovery of an effective WASH strategy could hasten control, but even when known to be effective against a disease, WASH does not work rapidly. Chlamydial vaccines might prove useful but are still in the early stages of development. The real hope may be more frequent MDA.^4^ Twice yearly or even more frequent MDA may be able to eliminate infection in the most severely affected areas. If so, by 2030 trachoma could not only be eliminated as a public health concern, it could be eradicated.
